# The earliest domestic cat on the Silk Road

**DOI:** 10.1038/s41598-020-67798-6

**Published:** 2020-07-09

**Authors:** A. F. Haruda, A. R. Ventresca Miller, J. L. A. Paijmans, A. Barlow, A. Tazhekeyev, S. Bilalov, Y. Hesse, M. Preick, T. King, R. Thomas, H. Härke, I. Arzhantseva

**Affiliations:** 1 Central Natural Science Collections, Martin Luther University Halle-Wittenberg, Domplatz 4, 06108 Halle (Saale), Germany; 2 Department of Archaeology, University of Exeter, Laver Building, North Park Road, Exeter, EX4 4QE UK; 3 Department of Archaeology, Max Planck Institute for the Science of Human History, Khalaische Str. 10, 07745 Jena, Germany; 4 Department of Anthropology, University of Michigan, 101 West Hall, 1085 S. University Ave., Ann Arbor, MI 48109-1107 USA; 5 Graduate School of Human Development in Landscapes, Christian-Albrechts-Universität Zu Kiel, Leibnizstrasse 3, 24118 Kiel, Germany; 6 Institute for Prehistoric and Protohistoric Archaeology, Archaeological Stable Isotope Laboratory, Christian-Albrechts-Universität Zu Kiel, Johanna-Mestorf-Strasse 2-6, 24118 Kiel, Germany; 7 Faculty of Mathematics and Natural Sciences, Institute for Biochemistry and Biology, University of Potsdam, Karl-Liebknecht-Str. 24-25, 14476 Potsdam, Germany; 8 Department of Genetics and Genome Biology, University of Leicester, University Road, Leicester, LE1 7RH UK; 9 School of Science and Technology, Nottingham Trent University, Clifton Lane, Nottingham, NG11 8NS UK; 10 Research Centre for Archaeology and Ethnography, Korkyt-Ata State University of Kyzylorda, 29A Aiteke bie str., 120014 Kyzylorda, Kazakhstan; 11 Department of Archaeology, Al-Farabi Kazakh National University, 71 al-Farabi Ave, 050040 Almaty, Kazakhstan; 12 School of Archaeology and Ancient History, University of Leicester, University Road, Leicester, LE1 7RH UK; 13 Department of Medieval Archaeology, University of Tübingen, Schloss Hohentübingen, 72070 Tübingen, Germany; 14 Centre for Classical and Oriental Archaeology, Higher School of Economics, House 3-L, Staraya Basmannaya Ulitsa 21/4, Moscow, Russia 105066; 15 Institute of Ethnology and Anthropology, Russian Academy of Sciences, Leninsky Prospekt 32a, Moscow, Russia 119334

**Keywords:** Stable isotope analysis, Archaeology, Archaeology, Genetic variation

## Abstract

We present the earliest evidence for domestic cat (*Felis catus* L., 1758) from Kazakhstan, found as a well preserved skeleton with extensive osteological pathologies dating to 775–940 cal CE from the early medieval city of Dzhankent, Kazakhstan. This urban settlement was located on the intersection of the northern Silk Road route which linked the cities of Khorezm in the south to the trading settlements in the Volga region to the north and was known in the tenth century CE as the capital of the nomad Oghuz. The presence of this domestic cat, presented here as an osteobiography using a combination of zooarchaeological, genetic, and isotopic data, provides proxy evidence for a fundamental shift in the nature of human-animal relationships within a previously pastoral region. This illustrates the broader social, cultural, and economic changes occurring within the context of rapid urbanisation during the early medieval period along the Silk Road.

## Introduction

Burials of domestic and wild cats remain a rarity in the archaeological record, especially in comparison to dogs, which are recovered so frequently that they have their own depositional typologies^1–5^. Finds of individual, articulated animals in archaeological contexts, particularly those exhibiting osteopathologies, are ideal for a social zooarchaeological approach which encourages the investigation of human animal interaction as a facet of social and cultural structures beyond productive capacity^6^. This type of integrated social-cultural approach in archaeology has largely been utilised in pre- and protohistoric contexts in which there are limited written sources in order to access and interpret the worldview of past cultures. However, there is value in utilising this framework in historical contexts as some animals, such as the domestic cat, are often not frequently and explicitly mentioned in the written record. Such animals act as signifiers of changing cultural attitudes and evidence of human-mediated species dispersal that accompanied expanding human connectivity through the end of the first millennium CE^7, 8^. The unique and rare find of the remains of a small felid, found in the medieval urban center of Dzhankent in Kazakhstan, is ripe for this approach. Below, we combine multiple lines of scientific enquiry to: explore the ancestry of this cat and ascertain its domestic status; characterise its diet in comparison to other fauna; and reconstruct its life history. Thus, this osteobiography reflects changing human attitudes towards animals that are part of bigger social and cultural changes taking place along the medieval Silk Road.

**Figure 1:**
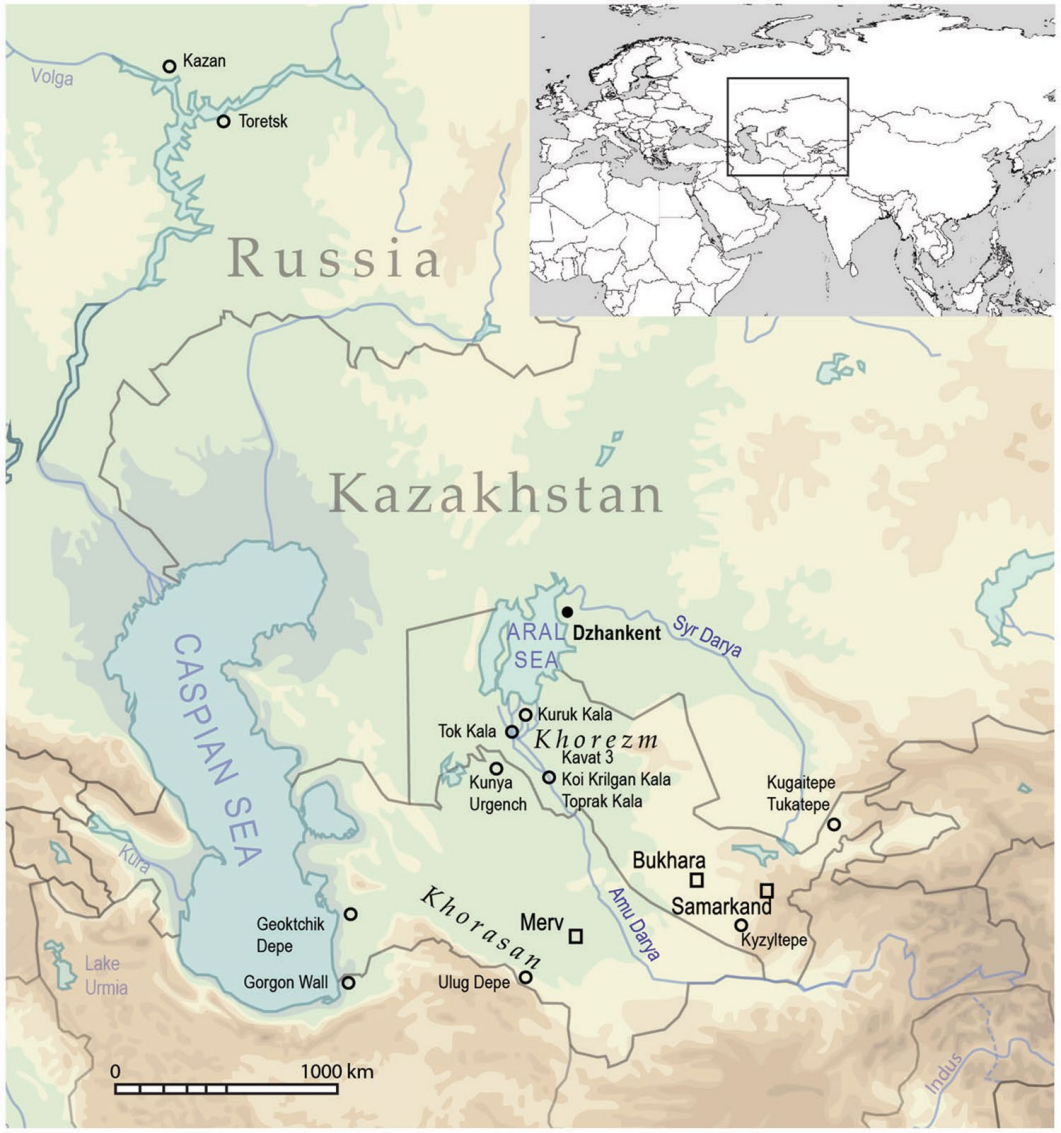
Location of Dzhankent and other archaeological sites mentioned in the text. Sites marked with squares are contemporaneous urban centres. Map generated by authors in qGIS (www.qgis.org) and Adobe Illustrator (www.adobe.com); basemap data Openstreet (www.openstreetmap.org), adapted under a CC BY-SA 2.0 license (https://creativecommons.org/licenses/by-sa/2.0/), boundaries vector file DCW (https://worldmap.harvard.edu/data/geonode:Digital_Chart_of_the_World?fbclid=IwAR2aFoqB1oWgay4fFXp0IifLZvsJobf0zaMYhYJo1H7TD5JHrX-RmaAA_1g).

In the semi-arid and arid steppe of Central Asia, particularly in modern day Kazakhstan, domestic cats were not widespread before the colonial period of the eighteenth and nineteenth centuries. There are no published archaeological remains of domestic felines from any prehistoric archaeological sites in the Kazakh steppe^9–12^ despite the fact that there is increasing evidence for sedentary settlements and agricultural activity dating from the Late and Final Bronze Age within this region which would encourage the presence and utility of commensal rodent hunters such as cats^13–15^. Along the southern border of the Central Asian steppe, in Uzbekistan and Turkmenistan, single bones from felines of uncertain domesticated status have been found in Bronze Age urban contexts, such as at the sites of Kavat 3 and Ulug Depe, as well as in Iron Age contexts from Geoktchick Depe and Kyzyltepe (Fig. 1)^16, 17^. Remains of small felines are similarly sparse throughout the Archaic and Antique periods in this region (fourth century BCE to eighth century CE) and only single bones were found at urban sites such as at Kugaitepe, Tukatepe, Tok-Kala, Toprak-Kala, Koi Krilgan Kala, Kuruk Kala and at the Gorgon Gates^16–19^. Osteological remains of cats dramatically increase from 1500 CE, and are found in much greater numbers at the urban site of Kunya-Urgench^20^. Indeed, there is a similar paucity of feline remains in periods prior to 1000 CE to the north in modern day Russia, after which cat remains are more commonly found at sites such as Novgorod and Tver, north of Moscow, and closer to the steppe boundary in Kazan and Toretsk^21–23^.

Felines are most frequently found in archaeological contexts in this region not as osteological remains but as artistic ornaments, and feature prominently in the iconography of animal style art, typical of Iron Age steppe cultures of the late first millennium BCE in Kazakhstan, Tuva, and Mongolia. In particular, motifs of predatory felines in twisting poses found in metalwork and in body art were likely inspired by large cats with a natural range across Central Asia and Persia, such as the snow leopard (*Panthera unica*), leopard (*Panthera pardus*), Eurasian lynx (*Lynx lynx),* lion (*Panthera leo*), and caracal (*Caracal caracal*)^24–26^. Large feline predators were also depicted in the early first millennium CE in Persia, as seen on rhytons dating to the Parthian period (The Metropolitan Museum of Art, New York, Cat.: 1979.447) and much later in metal objects from the twelfth and thirteenth centuries CE^27^. Conversely, a number of smaller wild felids, such as the manul (*Otocolobus manul*), jungle cat (*Felis chaus*), sand cat (*Felis margarita*), Chinese mountain cat (*Felis bieti*), and the local subspecies of wild cat, (*Felis lybica ornata*), are not similarly represented in material cultures of the region and there are no published zooarchaeological finds of these taxa. Furthermore, the palaeogeograhic dispersal of these species within the medieval period remains uncertain^25^. The earliest historical mention of domestic cats originates from Persia and dates to the sixth century CE, when it was specifically noted that women kept cats as pets, dying their fur, adorning them with jewellery, and letting them sleep in their beds. In contrast, historians note that Zoroastrians from the same region held cats in contempt, associating them with the dark magic of the jinn and condemning the pungent odour of their urine^28^. These widely divergent attitudes in multicultural and cosmopolitan urban spheres of Persia, Khorasan, and Khorezm, indicates that domestic cats were widespread within human settlements and not just valued for their economic value as mousers, but also anthropomorphised and kept as pets.

Urbanisation is not a feature commonly associated with steppe societies, although there is evidence for dense conurbations in the central semi-arid steppe, dating from the Late and Final Bronze Age (c. 1500 BCE), such as Kent^29^. Steppe cultures are often characterised by a relatively dispersed settlement pattern and are largely agro-pastoralist with a component of mobility associated with seasonal resource exploitation, particularly in arid ecologies and have a distinct lack of felid remains. Instead, domestic cats are generally associated with urban agricultural contexts that proliferated across the medieval world, as they prey upon rodent and bird commensals associated with grain stores, and it is puzzling that these animals have not been found across the steppe as agricultural activity certainly was occurring, albeit at a small scale, throughout prehistorical and historical contexts^7, 15^. Larger and more permanent settlements are characteristic of the oases and delta of the Amu Darya which belong to the Persian cultural sphere. Small defensive fortifications of the Dhzety-Asar culture first appear in the delta of the Syr Darya in the Late Iron Age/Antique periods (first to seventh centuries CE). Dzhankent was an early medieval city and one of three ‘marsh-towns’ now shown to have been founded in the seventh century CE^30^. It was located in the Syr Darya delta on a palaeochannel and close to a former inlet on the northeastern shore of the Aral Sea. The fortification of this urban site, increasing population density, and craft activities have been dated to the late ninth or tenth century CE, which is contemporary to the increasing proliferation of urban sites across Eurasia^31^. The origin of the trend of increasing density of human settlement remains uncertain, but is probably connected with intensifying long-distance trade between Khorezm and populations to the north and northwest in modern day Russia along branches of the Silk Road^31^.

Recent excavations have shown that the city of Dzhankent was a walled settlement with planned rectangular layout, with a citadel located in the northwestern corner^32^. Material culture dating from the seventh to twelfth centuries CE includes elements of intrusive nomadic Oghuz culture from the eastern steppes, the previous Dhzety-Asar culture, and neighbouring Khorezm, implying a mixture of nomadic and sedentary ‘urban’ cultures with ties to both the steppe to the north and urban cultures to the south. The Oghuz are reported as controlling the city and ruled this region until the eleventh century CE, when a significant proportion of the ruling classes were pushed south and reformed as the Seljuk Turks, conquering Persia and eventually most of the Middle East in the twelfth century^33^.

### Zooarchaeological recovery and analysis

**Figure 2:**
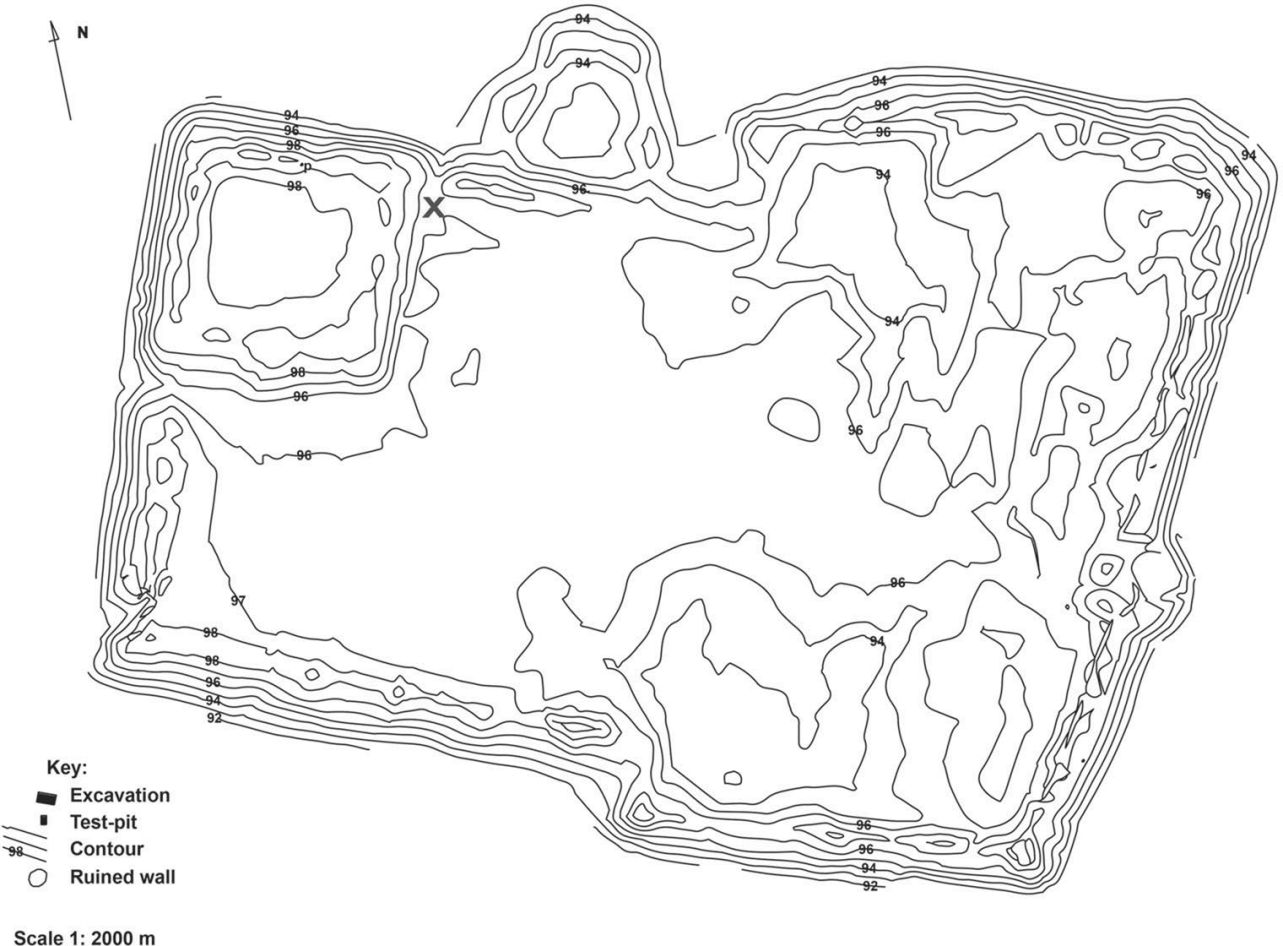
Site Plan of Dzhankent with location of recovered osteological material marked by a red cross.

**Figure 3:**
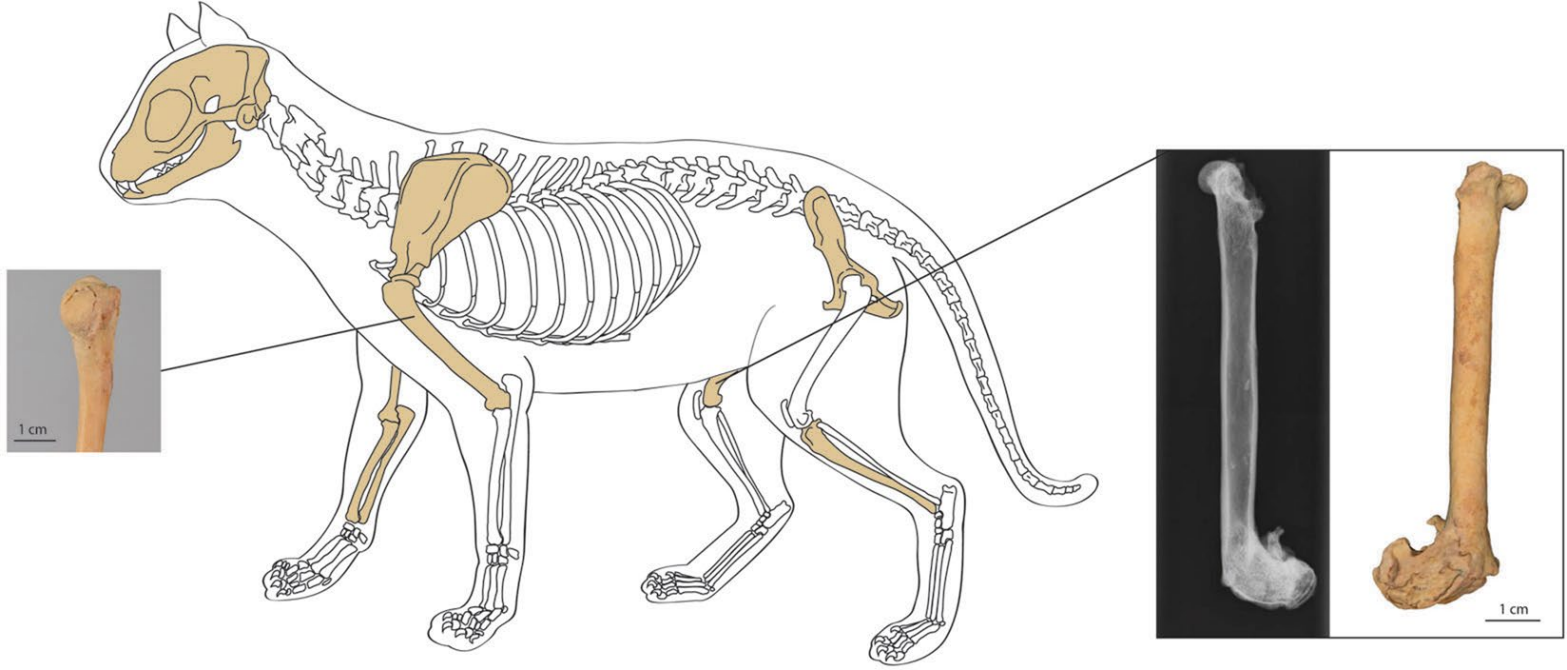
Skeletal elements present shaded; all recovered elements have recorded palaeopathologies. Left inset: Close up of articular fracture on left humerus. Right inset: Xray and view of 3D model of right femur.

A partially articulated cat skeleton was found in a midden context at the juncture of the citadel and city walls in the fill of an abandoned house, specifically in Trench 2, Quadrant 103/99, 446–487 cm below the top of the preserved citadel wall (Fig. 2)^32^. The context was characterised by abundant animal and fish bone within a loose light brown clayey silt matrix. A complete skull and left and right mandible, as well as a left scapula and humerus, right humerus, ulna and radius, left pelvis, right femur, left tibia, and four vertebrae were recovered from a single individual feline (Fig. 3, Supplementary Table 1). The bones were well preserved, with little evidence for weathering or post-mortem modification. These remains are categorised as an expedient burial as there is no clear grave cut and little evidence of ritual or sacred function^2^. Radiocarbon dating of the cat femur produced an uncalibrated date of 1,171 ± 15 BP (GrM-12987), with a 95% calibrated date range of 775–940 CE^34–36^.

Using epiphyseal fusion and tooth eruption, this cat was at least 58 weeks old at death; however, the presence of age-related osteological pathologies across the skeleton indicate that this cat was likely older than this lower limit estimate^37, 37, 38^. Comparison of greatest length data of limb bones against published contemporaneous domestic cats from Russian archaeological contexts, modern British domestic and wildcats with sexing data, as well as published standards for European domestic and wild cats indicate that this animal was a similar size to an intact (uncastrated) male domestic cat, but smaller than a European wildcat (Supplementary Table 2)^23, 39, 40^. A macroscopic examination of morphological characteristics and comparison to modern comparative zooarchaeological collections housed at the School of Archaeology and Ancient History at the University of Leicester as well as regionally specific comparative literature was inconclusive for identifying this specimen to species as extensive pathologies obscured qualitative discriminative features^41, 42^.

### Genetics: identifying ancestry

Recent genetic research has indicated that all modern domestic cat populations originate from the African wildcat, *Felis lybica lybica*^7, 8, 25, 43, 44^. However, disentangling the genetic structure of domestic cats and wildcats is challenging, as there is ongoing hybridisation between lineages^8, 43, 45^. Central Asian wildcats (*F. l. ornata*) have been reported to have a high frequency of African wildcat mitochondrial haplotypes (up to half of the sampled cats^43^), making mitochondrial DNA unsuitable for ancestry determination. In contrast to mitochondrial DNA, nuclear data has revealed a clearer population structure, with the wildcats and domesticated cats forming distinct nuclear clades^8, 43^. Therefore, we employed genetic analysis of nuclear DNA to explore the ancestry of the Dzhankent specimen.

**Figure 4:**
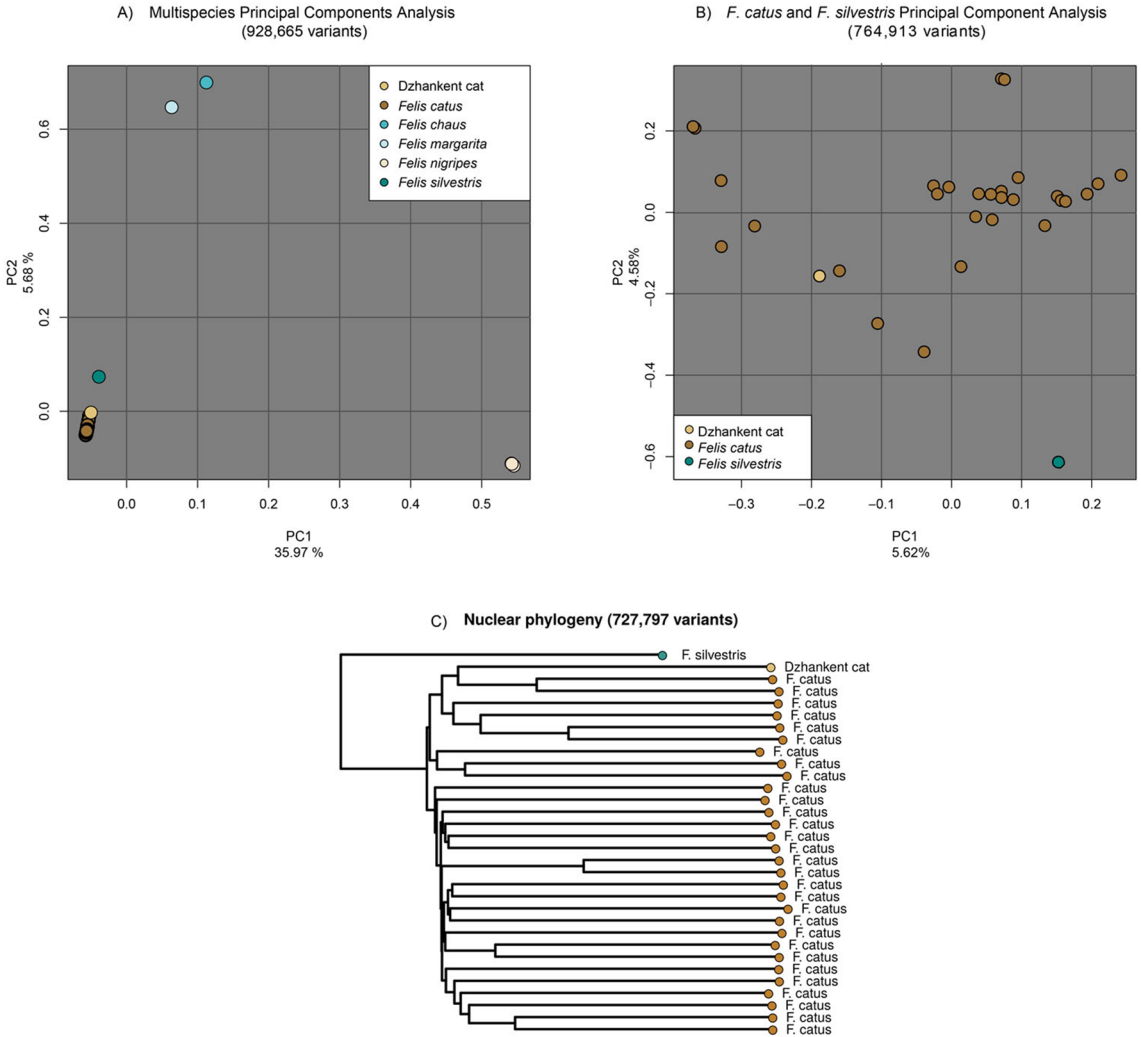
Principal component analysis of multi-species genomic data (**A**) and domestic and wildcat genomic data (**B**; Supplementary Table 3), as well as a neighbour-joining phylogeny of the nuclear genomes of domestic cats, the Dzankent cat, and a wildcat (**C**). Analyses are based on 928,665 (**A**), 764,913 (**B**) and 727,798 (**C**) variable positions after filtering (see Methods). Axis labels in A and B indicate the percentage of variance explained by each component. The neighbour-joining phylogeny is rooted with the black-footed cat (*F. nigripes*) as outgroup (not shown). Colours reflect the (sub)species as indicated in the legends.

DNA was extracted from the petrous bone and converted to single-stranded sequencing libraries using dedicated ancient DNA protocols^46–48^. Approximately 52 M reads were generated, of which 48% failed to pass the minimum length filter (< 30 bp). Of the remaining reads, 79% reads could be mapped to the domestic cat (*Felis catus*) reference genome^49^, suggesting a high endogenous content for the petrous bone of this specimen. This is in line with previous results from the petrous bones of humans^50^ and cave bears^51^. The average coverage for the autosomes is approximately 0.38 – 0.43x, and 0.22 × for the X-chromosome assembly: this reduced coverage for the X-chromosome is a strong indication that the Dzhankent individual was male. The mitochondrial haplotype of the Dzankent cat falls within a clade containing domestic cat (*F. l. lybica*) haplotypes (Supplementary Fig. 1), but this provides weak evidence of ancestry. To further investigate this, we carried out population clustering analysis based on low coverage nuclear genome data of the Dzhankent cat and publicly available datasets representing multiple cat species. This analysis shows a clear separation of taxa^8, 43^ (Fig. 4a; Supplementary Table 3), with the largest component representing more than 35% of the variation in the filtered data. The Dzhankent cat specimen clusters with the domestic cats (Fig. 4a). A second analysis of wild European wildcat (*F. silvestris*) and domestic cats (*F. catus*) shows the same clustering (Fig. 4b). Phylogenetic analysis of the whole nuclear genomes supports the monophyly of the domestic cats including the Dzhankent cat, which together form a sister group to the European wildcat genome (Fig. 4c).

Unfortunately, at the time of this project, no nuclear shotgun genome data was available from the Central Asian wildcat *F. l. ornata*, preventing a direct comparison between the modern Central Asian wildcat data and the ancient Dzankent cat. Previous studies of nuclear data (short tandem repeats and microsatellites^43^) collected from modern cats do suggest that the Central Asian wildcat is genetically distinct from the domesticated cat, as well as from other cat lineages. Under this assumption that Central Asian wildcats and domestic cats are genetically distinct at the whole genome level, the clustering of the Dzhankent cat with domestic cats in the principal component analysis suggests that the individual has a higher affinity with the population that was domesticated in the Near East. Long-distance transportation events have been suggested previously by the presence of *F. l. ornata* mtDNA haplotypes in Turkey (Ottoni et al., 2017), and it is possible that the Dzankent cat may have also been moved north from the Middle East.

It should be noted that by increasing the number of analysed genetic markers from a handful of markers in previous studies to several hundreds of thousands using whole nuclear genome data, future analyses may reveal extensive admixture and incomplete lineage sorting between domestic cats and other Asian wildcats. Comprehensive geographic sampling of Asian wildcats will thus be required to assess the genetic structure of domestic cats and the Asian wildcat, and conclusively verify the assignment of the Dzhankent cat to *F. l. lybica*/*F. catus.*

### Stable isotopes: feline diet, ecological context, and urban provisioning

**Figure 5:**
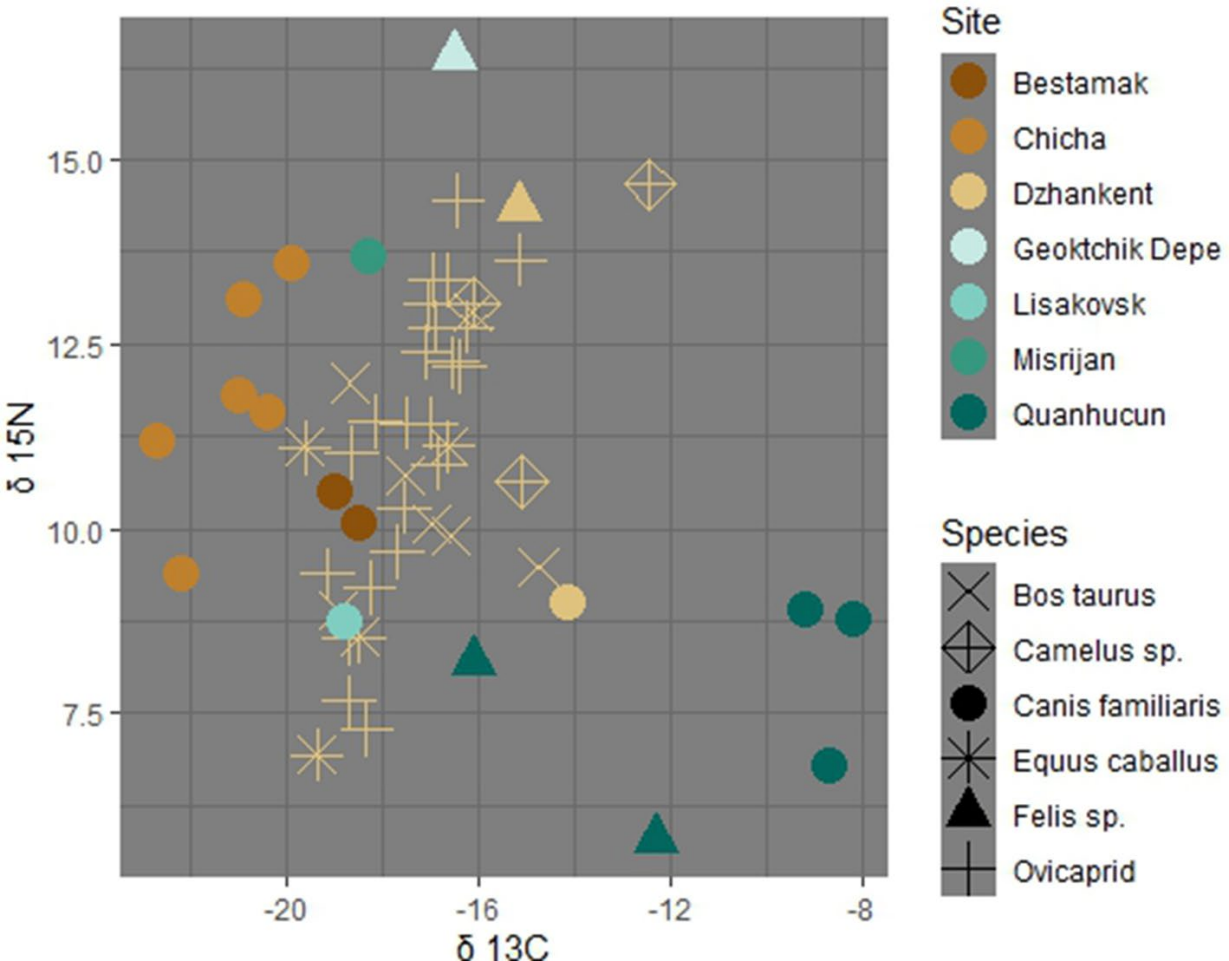
Scatterplot of stable isotope values of fauna from Dzhankent and other Asian archaeological sites.

Carbon and nitrogen stable isotopes have routinely been used to reconstruct human and animal diets in Eurasia^52–55^. There is also a growing body of evidence regarding the dietary intake of domesticated animals, specifically those that may have served as companions (such as dogs and cats) or eaten human refuse^56–58^. To clarify the dietary intake of the feline under study, we analyzed stable carbon (δ^13^C) and nitrogen (δ^15^N) isotopes of fauna (n = 34) from the site of Dzhankent and compared our results to previously published isotope values from the broader region (Fig. 5).

The cat was sampled at the medial fibula, which ossifies in the first months of life, and has a δ^13^C value of − 15.1‰ and a δ^15^N of 14.4‰, while the dog from the site has values of − 14.2‰ and 9.0‰, respectively^59^. Livestock (camels, cattle, sheep, goat, horses) from Dzhankent have a wide range of δ^13^C values, from − 19.6 to − 14.7‰ (average value of − 17.4‰) and 6.9 to 14.5‰ δ^15^N values (average value of 11‰). The wide range of isotope values for fauna from this site indicates that livestock exploited resources from varied ecologies near this oasis, which was located in a relatively arid region.

The cat had similar carbon isotope values, but higher nitrogen isotope values (by 5.4‰) than the dog from the same site. When compared with zooarchaeological remains of dogs and cats from across central Asia, the cat at Dzhankent was enriched in ^15^ N by more than 5‰, with the exception of a cat from Geotchikdepe (16.4‰) and dogs from Chicha (range from 9.4 to 13.6‰)^52, 56, 60, 61^. Large quantities of fish remains were recovered from the sites of Chicha and Geotchikdepe suggest that these largely carnivorous animals consumed aquatic taxa which enriched their nitrogen values. As fish remains were identified at Dzhankent for this period and the site was located on a palaeochannel in the delta of the Syr Darya, fish would have been readily accessible protein resource, and likely contributed to higher δ^15^N values^32^. Both the cat and dog from Dzhankent were depleted in δ^13^C relative to dogs from Quanhucun, a site which had archaeological evidence for millet, but enriched in δ^13^C relative to most livestock from Dzhankent. This suggests that both taxa were ingesting small amounts of C_4_ plant resources directly, such as millet, or consuming fauna which were subsisting on C_4_ plants. As the livestock from this site have only slightly lower δ^13^C values than the two carnivores, it is possible that the dog and cat ingested rodents or other commensal species which were subsisting on agricultural products.

### Palaeopathologies: evidence for human care

This individual possesses extensive pathologies found across nearly all recovered skeletal elements which were recorded macroscopically using a descriptive recording protocol^62, 63^ (Supplementary Table 1). Osteological pathologies encompassing four nosological categories were identified: trauma, joint disease, inflammation, and dental disease. Trauma was evident on both left and right sides of the body. An oblique fracture was apparent in the distal metaphysis of the right femur (Fig. 3), which was in the process of healing at the time death. The fractured halves united with slight medial deviation and proximal rotation, such that the distal condyles were re-positioned 135° to the shaft. Articular contour changes to the distal femur and enthesophytes at the proximal end of the bone could represent secondary responses to altered biomechanical loading on connective tissues. The left humerus had an articular fracture on the proximal articulation, extending distally and medially from the centre of the humeral head towards the articular margin. Osteophytes and enthesophytes present on the left humerus and scapula and contralateral humerus, radius, and ulna may again be adaptations to altered loadings, although such lesions on and around the joints can also be caused by age-related joint disease. The left pelvis exhibited lesions characteristic of hip dysplasia including the flattening of the craniodorsal acetabular border and marginal osteophytes^64^. Unfortunately, the absence of the left femur meant that the adjacent lesions could not be assessed. However, the left tibia presented a change in the orientation of the proximal articular surface, ankylosis of the proximal fibula and evidence for calcification of ligament/tendon attachments, which may be a remodelling response associated with hip dysplasia in the same limb. In addition to the traumatic lesions, periosteal new bone formation was observed on the right ulna and left tibia. Ossification of the connective tissue surrounding the shafts of long bones is an inflammatory response but is relatively non-specific and can arise as a result of localised trauma, inflammation in the surrounding soft tissues, or systemic disease, and therefore cannot be directly connected to the above traumas. Some of the enthesophytes might be directly or indirectly associated with traumatic insult, however there may also be an age component, since ossification of attachment sites can occur with age. Supportive evidence for an age of the cat that is greater than one year of age is provided by the presence of marginal osteophytes and articular porosity in the mandible and distal tibia, characteristic signs of degenerative joint disease.

Significant and extensive ante-mortem tooth loss of mandibular incisors and canines with root infilling and ante-mortem loss of the second and third maxillary premolars on the left side was observed. The mandibles also presented evidence of porosity, periosteal new bone formation on the labial side and feathered alveolar edges. Calculus was present on the left maxillary fourth premolar. Enthesophytes were observed along the attachment site for the masseter muscle, while osteophytes and articular contour changes were apparent in the temporo-mandibular joint. Radiographic examination of the mandibles indicated early bone loss in the furcation of the right P4 and furcations of the left premolars with a reduction in the alveolar crest. All of these are clear signs of early stage periodontitis which could be the cause of ante-mortem tooth loss^65^. There is no radiographic evidence of feline odontoclastic resorptive lesions or tooth resorption (TR) in the mandible and maxillary dental arch^66^.

Periodontal disease arises from escalation of inflammation of the gingiva, usually following the accumulation of bacteria-rich dental plaque, and is strongly influenced by diet, with a soft diet favouring plaque and calculus accumulation and thus gingivitis; however, it also increases in frequency and severity with age^67, 68^. Canine loss is correlated increasing age in cats and is also more prevalent in feral cat populations relative to domestic cats and may not have been solely due to periodontal disease, as TR has multiple aetiologies, and can also be caused by disturbances in vitamin D and calcium metabolism, although this is unlikely given this individuals diet^69, 70^. The presence of intact calculus suggests that a soft sticky food was a component of the cat’s diet in the weeks prior to death; research investigating the prevalence indicates that calculus deposition peaks at four weeks, after which calculus presence decreases^71^.

The pathologies in the skeleton suggest that the cat from Dzhankent had a physically challenging life, as evidenced by three traumatic lesions: fractures to the distal right femur and articular surface of the left humerus, and dislocation of the left femur. While congenital hip dysplasia is required in the differential diagnosis of the latter, it occurs much less commonly in cats than dogs^64^. Whether the traumatic episodes occurred in the same event or independently is difficult to establish. Furthermore, the time between the trauma and death is hard to determine and healing time for fractures depends upon both biological factors, such as the age of the individual, associated traumas, and blood supply, as well as mechanical factors, such as stabilisation of the fracture site^72^. Certainly, the fracture to the distal right femur was still healing at the time of death as evidenced by the presence of lamellar and woven bone within the callus. The angle of ossification of the fracture indicates that this animal may have been restrained, as there is little evidence for torsion at the fracture site.

A fracture to all three parts of the skeleton occurring in the same event is perhaps only conceivable if the cat experienced a fall from a considerable height. Irrespective of the cause, some of the lesions observed at and around the joints in the skeleton, such as new bone formation at ligament and tendon attachment sites and the morphological changes to the proximal tibia, may reflect secondary remodelling to compensate for altered biomechanical stresses or direct stress experienced by connective tissues, such as torn ligaments. The combined effect of these lesions would have been to considerably limit the mobility of this animal prior to its death, hindering its ability to capture prey, flee to shelter, and to fight. Inflammation and tooth loss would have caused discomfort and potentially affected the cat’s ability to catch and consume food as well as defend itself^73^. The presence of both calculus on the maxillary fourth premolar as well as additional dental pathologies and isotopic data indicates that our cat likely had a soft diet that was high in protein which was consumed during bone ossification and in the weeks prior to its death.

## Discussion

This individual cat from Dzhankent has pushed back the date of the first archaeologically domesticated cats in this region by a millennium to 775–940 cal CE. This male adult cat likely descended from a Middle Eastern population of domestic cats, although comparative modern genetic data from regional wildcats is required to confirm this ancestry. Both stable isotope and dental palaeopathological data indicate this animal had access to a high protein diet from an early age up until the final weeks prior to death, which most likely caused gum disease, a common affliction in modern domestic cat populations. The extensive paleopathologies across the skeleton clearly demonstrate the high probability of human pet keeping behaviours which contributed to this individual’s survival. This cat lived through a number of traumas and would have had limited chances for survival without human intervention, as it would have had trouble hunting with both physical impairments and loss of canines. Furthermore, the angle of ossification of the femoral fracture indicates that this animal may have been restrained during the healing process, which was likely only possible with human care and attention.

There was a steep increase in the number of domestic felines in European and Middle Eastern contexts at the end of the first millennium CE^16–19, 21–23, 74, 75^. This has been tied to the need to control commensal activity in the proliferation of new urban settlements and to increasing international trade that connected the Islamic world with northern Europe, the Mediterranean and Northern Africa^7, 76^. Therefore, the appearance of a domestic feline at Dzhankent suggests that this urban settlement was integrated with a network of trade and commerce, despite its relatively isolated location on the steppe. Archaeological and historical evidence indicates that Dzhankent participated in global trade networks, most likely taking part in the major north/south axis of the slave trade, which linked the slave markets of the caliphate in the Amu Darya delta with two major regions where slaves were captured: the steppe to the east (Bilad al-Atrak) and eastern and northern Europe (Bilad al-Saqaliba) via the major slave route originating in Bulgar^31–33,77^.

The evidence for human care of this animal indicates a shift in trajectory in the human-animal relationships typically found on the Central Asian steppe. The city of Dzhankent was unusual, as it was ruled by the Oghuz, a people that originated from the rural hinterlands of the Central Asian steppe and had their origins as a nomadic pastoralists, who have no documented archaeological evidence for keeping domestic cats. This cat’s osteobiography demonstrates deep and pervasive cultural links to other regions, particularly to the south to the regions of Khorezm, Khorasan and Persia, where pet keeping and feline anthropomorphisation were widespread practices by the arrival of Islam in the eight century CE^28^. Throughout the early medieval Islamic world, cities proliferated as both international trade and increasing degrees of social and cultural sophistication were celebrated^77^.

While the Oghuz elite would have contributed a strong pastoral worldview to the culture of Dhzankent pet keeping and tolerance of small felids is an urban idea, one that is typically associated with permanent agricultural settlements. Therefore, evidence for human kindness and care suggests the presence of a cosmopolitan population which harboured a diversity of worldviews on animals. While it is not possible to determine whether this cat was part of a local population of cats or another import that arrived via international trade, it is clear that the people of this town had a different perspective on pet keeping than previous steppe populations. They carefully cared for and tended to this animal throughout its life, providing high quality food and medical care. Thus, this small cat is not only the earliest known domestic cat on the Silk Road, but also evidence of the complex interface between nomadic and urban cultural worldviews in a rapidly globalising world.

## Methods

### Methods: zooarchaeological recording

Metrics were taken according to zooarchaeological standards (Supplementary Table 2)^78^. Three dimensional models of the bones were recorded using a DSLR Canon 80D, an automated turntable, and Autodesk Recap® software and these models are available in an open access repository Zenodo (10.5281/zenodo.3490934)^79^. The bones were subjected to digital radiographic examination using an Xograph DRagon mobile x-ray unit at the University of Leicester (60 KvP; 6.40 mAs, 0.025 s).

### Methods: genetic analyses

#### Laboratory procedures

The petrosal part of the temporal bone was sampled from the Dzhankent cat specimen through one of the auditory channels, as this skeletal element has been found to contain high purity DNA in other mammals^50, 51^. Extraction and high-throughput sequencing libraries were performed following strict decontamination procedures in dedicated ancient DNA facilities at the University of Potsdam. Two independent DNA extractions were performed following a protocol specifically developed for the recovery of short fragments generally found in ancient samples^46^. For each extract, one Illumina sequencing library was prepared according to a single-stranded library preparation method developed for ancient DNA^47, 48^. UDG/Endonuclease VIII treatment was performed for all libraries to excise uracils that arise from cytosine deamination (except the uracils occurring as terminal nucleotides) that damages DNA over time^80^. Optimal library amplification cycles were estimated using qPCR, and libraries were subsequently indexed following a dual indexing protocol to detect and remove chimeric sequencing artefacts and reads^81^. Sequencing libraries generated from the extraction blanks required five cycles more than the sample libraries, indicating that there was at least 32-fold less genetic material in the blanks, supporting the absence of detectable contaminant DNA. Sequencing was performed on the Illumina NextSeq 500 platform using a 75 bp single-end strategy^82^.

#### Sequence data processing

Raw sequencing reads were trimmed of the adapter sequence using cutadapt v1.12^83^, using default parameters and a minimum length filter of 30 bp. Remaining sequence reads were mapped to the domestic cat genome v6.2^49^ as well as the cheetah reference genome *Acinonyx jubatus v1*^49^, as using an intraspecific reference genome as been shown to potentially introduce biases in downstream analyses^84^, using the Burrows–Wheeler Aligner (BWA v0.7.8^85^) ‘aln’ and ‘samse’ algorithm with default parameters. Samtools v0.1.19^86^ was used to filter reads with low mapping quality (MQ < 30) and suspected PCR duplicates. Average coverage was calculated using the GenomeAnalysisTK v2.8 (https://software.broadinstitute.org/gatk/). Sex determination was performed by mapping the ancient cat sequences to the domestic cat assembly, and then comparing the average coverage for autosomal regions to the coverage of the X-chromosome assembly. Nucleotide misincorporation patterns indicating typical ancient DNA damage were checked to validate sequence authenticity using mapDamage v2.0.7^87^ (Supplementary Fig. 2).

In order to compare the nuclear data from the Dzhankent cat to publicly available genome data, 37 shotgun sequencing datasets were selected from NCBI to reflect a range of breeds and species (Supplementary Table 3). Data was downloaded from the European Nucleotide Archive FTP server, and subsampled to 50–100 million reads using seqtk v1.2-r101 (https://github.com/lh3/seqtk) (Supplementary Table 3). Adapter sequences were trimmed using Skewer v0.2.2^88^, and overlapping reads were merged using flash v1.2.10^89^ using default parameters for both. Mapping of the published data was performed using the same pipelines as described above, except for using BWA ‘sampe’ instead of ‘samse’. For mitochondrial DNA analysis, reads were mapped to the domestic cat mitochondrial genome (GenBank accession number FCU20753^90^); following the same procedure as the nuclear genome, except for duplication removal which was performed taking both mapping coordinates into account (‘MarkDupsByStartEnd.jar’ (https://github.com/dariober/Java-cafe/tree/master/MarkDupsByStartEnd). For the phylogenetic analysis of mitochondrial DNA, 233 short sequences (ND5 & ND6 genes)—mainly from domestic cat and wildcat—were downloaded from GenBank and aligned with the Dzhankent cat, and the published individuals included in the genome analysis (Supplementary Table 3 & 4). Columns with gaps or missing data were removed, resulting in a 2,363 bp alignment of 264 taxa. A median-joining network (Supplementary Fig. 1) was generated using PopArt v1.7.0^91^, based on 214 segregating sites and 89 parsimony-informative positions.

#### Population clustering and phylogenetic analyses

Population structure was tested based on the genotype likelihood method (major/minor allele) in ANGSD v0.914^92^ using random base sampling. Data was filtered for low base quality (-MinQ 30) and mapping quality (-minMapQ 30). Only reads mapping to cheetah scaffolds over 1 Mb were used in this analysis. As -MaxDepth parameter, we applied the 95th percentile of the global coverage as recovered using ANGSD doCounts. The analysis was restricted to transition sites (-rmTrans 1), and to sites where all individuals were covered by at least one read (-minInd). Singletons were excluded by applying a minimum allele frequency filter of 2/N of individuals. Two analyses were performed: one including only domestic cat and wildcat data, and one multi-species analysis with three additional cat species (*F. margarita*, *F. nigripes* and *F. chaus*). Principal component analysis of the resulting data was performed in R v3.4.4 and v3.5.1^93^. The nuclear phylogeny was estimated by calculating a pairwise distance matix using indentical data filters for all domestic and wildcat data, including a representative of *F. nigripes* to serve as outgroup. A rooted neighbour-joining tree was then calculated using the nj function in the R package ape^94^.

### Methods: isotopes

Carbon and nitrogen isotope samples were prepared in the Archaeological Stable Isotope Laboratory (ASIL) at Christian Albrechts University at Kiel. Samples of bone weighing ~ 0.5 g were demineralized in 0.5 M EDTA (pH 8.0) with a change of acid every other day until collagen pseudomorphs were translucent and flexible. The resulting collagen pseudomorpha were rinsed in distilled water seven times, with an overnight soak on to remove residual EDTA solution, and then rinsed a further eight times^95^. The samples were then rinsed in 0.1 NaOH to remove humic acids and rinsed again five times in distilled water. The collagen samples were freeze-dried and weighed for analysis. Stable carbon (δ^13^C) and nitrogen (δ^15^N) isotope analysis was undertaken at the Boston University Stable Isotope Laboratory using a EuroVector Euro EA elemental analyser coupled with a GVI IsoPrime in continuous flow mode. Analytical error was 0.1‰ and 0.2‰ for δ^13^C and δ^15^N, respectively. Isotopic values are reported in permil (‰) relative to the Vienna Pee Dee Belemnite (VPDB) standard for δ^13^C and atmospheric nitrogen (AIR) for δ^15^N. Collagen quality was assessed using %C, %N, and C:N ratios^96, 97^.

## Supplementary information

Supplementary file 1 (PDF 1531 kb)

Supplementary file 2 (XLS 119 kb)

## Data Availability

Raw sequence data has been deposited in the European Nucleotide Archive (Study Accession Number: PRJEB38002). The consensus mitochondrial genome sequence is available from GenBank (Accession number: MT499915). Three dimensional models of postcranial bones and metrical data are available at Zenodo (10.5281/zenodo.3490934).
