# Supplementary material for: The earliest domestic cat on the Silk Road
Source: Sci Rep. 2020 Jul 9;10:11241. doi: 10.1038/s41598-020-67798-6 (PMC7347622; doi:10.1038/s41598-020-67798-6)
Supplement: Supplementary file 1 — (PDF 1531 kb) [file 41598_2020_67798_MOESM1_ESM.pdf]

The earliest domestic cat on the Silk Road

Haruda, A*[1,2]; Ventresca Miller, A[3,4,5,6]; Paijmans, J.L.A[7,8]; Barlow, A.[9], Tazhekeev, A[10]; Bilalov, S[10,11]; Hesse, Y.[7]; Preick, M.[7]; King, T[8,12]; Thomas, R[12]; Härke, H[13,14]; Arzhantseva, I[14,15]

[1] Central Natural Science Collections, Martin Luther University Halle-Wittenberg, Domplatz 4, Halle (Saale) 06108, Germany
[2] Department of Archaeology, University of Exeter, Laver Building, North Park Road, Exeter, EX4 4QE, U.K.
[3] Department of Archaeology, Max Planck Institute for the Science of Human History, Khalaische Str. 10, Jena, 07745, Germany
[4]Department of Anthropology, University of Michigan, 101 West Hall, 1085 S. University Ave. Ann Arbor, Michigan, 48109-1107 U.S.A.
[5] Graduate School of Human Development in Landscapes, Christian-Albrechts-Universität zu Kiel, Leibnizstrasse 3, 24118 Kiel, Germany
[6] Institute for Prehistoric and Protohistoric Archaeology, Archaeological Stable Isotope Laboratory, Christian-Albrechts-Universität zu Kiel, Johanna-Mestorf-Strasse 2-6, 24118 Kiel, Germany
[7] University of Potsdam, Faculty of Mathematics and Natural Sciences, Institute for Biochemistry and Biology, Karl-Liebknecht-Str. 24-25, Potsdam, 14476, Germany
[8] Department of Genetics and Genome Biology, University of Leicester, University Road, Leicester, LE1 7RH, U.K.
[9]School of Science and Technology, Nottingham Trent University, Clifton Lane, Nottingham NG11 8NS, UK
[10]Research Centre for Archaeology and Ethnography, Korkyt-Ata State University of Kyzylorda, 29A Aiteke bie str., 120014, Kyzylorda, Kazakhstan
[11] Department of Archaeology, Al-Farabi Kazakh National University, 71 al-Farabi Ave., Almaty, 050040, Kazakhstan
[12] School of Archaeology and Ancient History, University of Leicester, University Road, Leicester, LE1 7RH, U.K.
[13] Department of Medieval Archaeology, University of Tübingen, Schloss Hohentübingen, 72070 Tübingen, Germany
[14] Centre for Oriental and Classical Archaeology, Higher School of Economics, House 3-L, Staraya Basmannaya Ulitsa 21/4, Moscow 105066, Russia
[15] Institute of Ethnology and Anthropology, Russian Academy of Sciences, Leninsky Prospekt 32a, Moscow, 119334, Russia

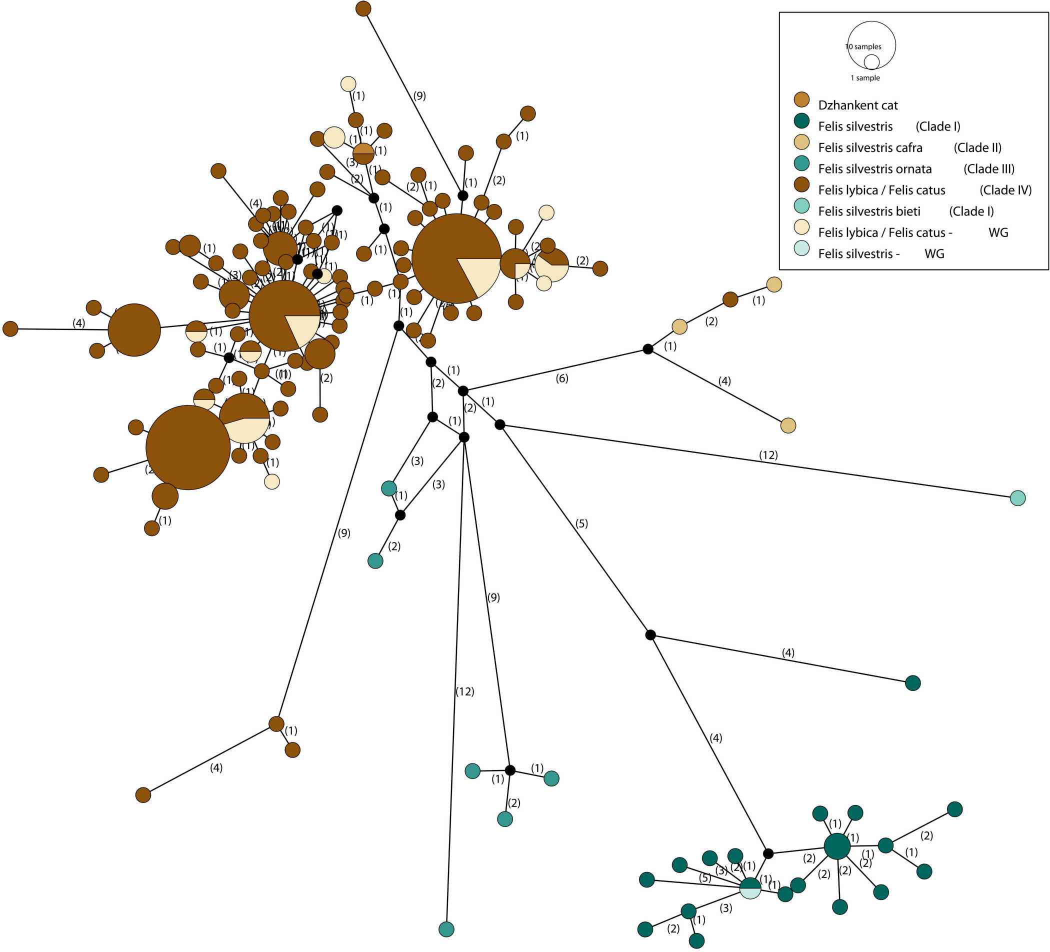

Supplementary Figure 1: Median-joining mitochondrial haplotype network including the Dzhankent cat, individuals used for nuclear analysis (Supplementary Table 3) and 233 additional published sequences (Supplementary Table 4), totalling to 264 taxa.

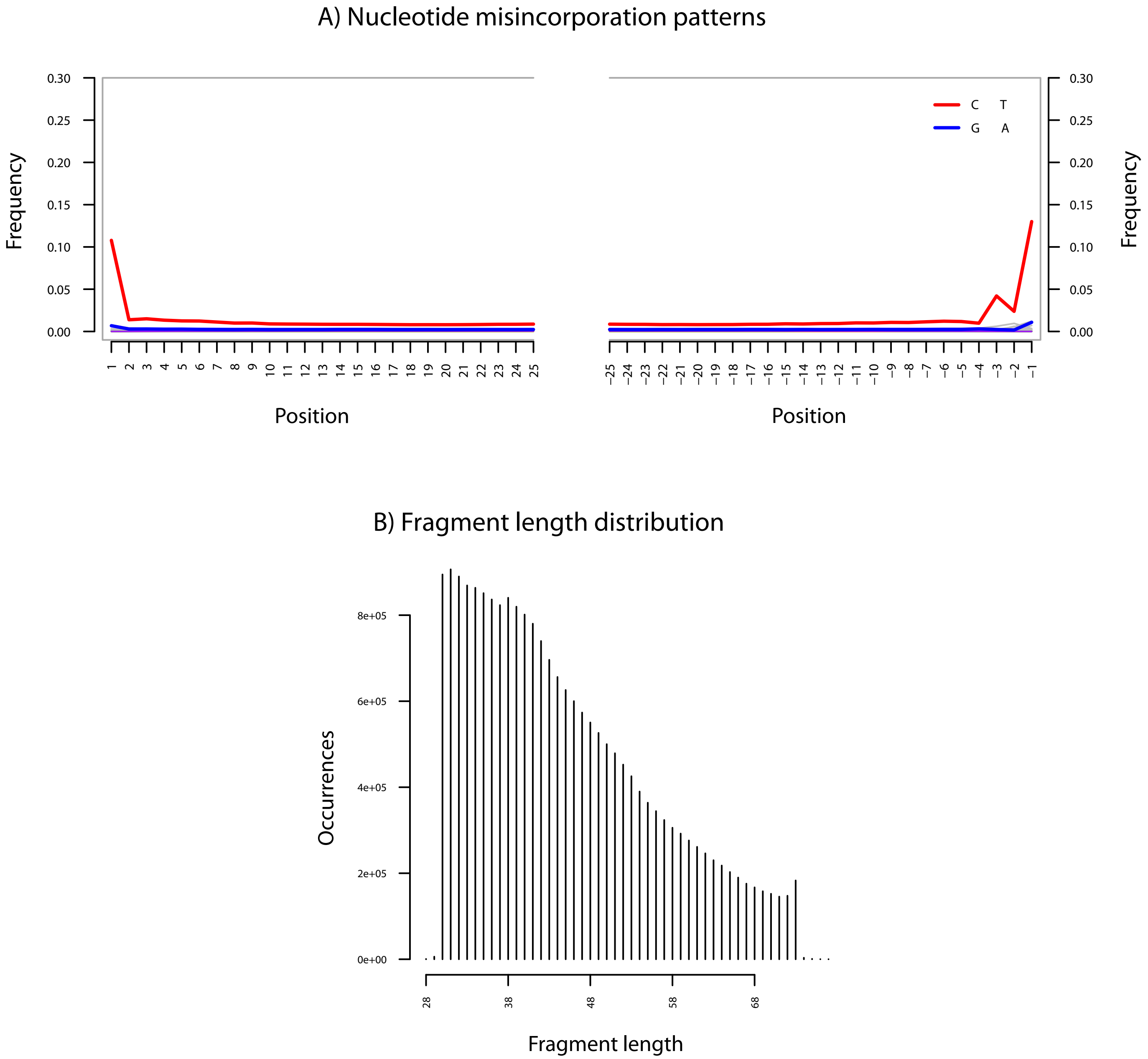

Supplemetary Figure 2: Assessment of ancient DNA authenticity. Fragment misincorporation patterns (A) estimated by mapping the Dzhankent data to the reference genome assembly of the domestic cat. The left and right panels show the frequencies of C to T (red) and G to A (blue) misincorporations for the the 5′ and 3′ read ends, respectively, with nucleotide position numbered sequentially on the x axes. The increase in C to T substitutions around the terminal nucleotides are consistent with cytosine deamination and support the authenticity of the data. The distribution of fragment lengths (B) indicate advanced DNA fragmentation with a median length of 42 bp, consistent with expectations for ancient DNA further supporting the authenticity of the data.
